# The Urinary Microbiota and the Gut–Bladder Axis in Bladder Cancer

**DOI:** 10.3390/ijms262110558

**Published:** 2025-10-30

**Authors:** Usman Akhtar Butt, Daniela De Biase

**Affiliations:** Department of Medico-Surgical Sciences and Biotechnologies, Sapienza University of Rome, Corso della Repubblica 79, 04100 Latina, Italy; u.akhtarbutt@gmail.com

**Keywords:** bladder microbiota, bladder cancer, gut-bladder axis, microbial metabolites

## Abstract

The human bladder hosts a resident, low-biomass microbial community (urobiota) that has only become the subject of intense investigation in the last 15 years. The advantages that the urobiota may confer to the bladder, in contrast to the microbiota of other mucosal sites, remain to be elucidated. Alterations in the urobiota have been associated with various pathological urogenital conditions, including urinary tract infections (UTIs) and recurrent UTIs. A potential link between bladder cancer (BC), the ninth most common human cancer by incidence worldwide, and a dysbiotic urobiota is still unclear and represents an emerging field of study. In this review, we focus on recent studies that not only analyzed the urobiota of BC patients using urine specimens to identify biomarkers and microbial signatures of the disease, but also monitored therapeutic responses to therapies. We also discuss novel techniques of culturing, such as culturomics, animal models of BC, and 3D organotypic models. Furthermore, we review studies on the gut–bladder axis which, though still limited, already suggest that diet- and gut-derived bacterial metabolites can influence BC progression and individual responses to therapy.

## 1. Introduction

The term microbiota refers to the communities of microorganisms residing in or on human body sites, whereas the term microbiome refers to the collective genetic content and its manifestation in time and space [1]. In the last decade, several studies have demonstrated the presence of a resident, specific, low-biomass urinary tract microbiota (urobiota), thus disproving the long-lasting belief that the urinary tract above the urethra is sterile and bacteria are found only in the presence of an infection [2,3,4]. The progress in our understanding of the role and composition of the urobiota is due to advancements in cultivation techniques and molecular techniques that have allowed the detection of microbial species in the urine of healthy subjects [5,6,7,8]. The urobiota has been shown to play an important role in protecting against infections and maintaining homeostasis, i.e., it supports the urinary tract epithelium, assists the immune system, and influences neurotransmission [8,9,10]. Together with the host environment, the urobiota constitutes a micro-ecological niche, the disruption of which may contribute to disease, though our understanding remains limited [9].

As for the origin of the urobiota, many questions are still open. It is suggested that a stable microbial community in the urinary tract forms within the first few years of life [11,12,13] and indeed microbial species have been detected in the urinary tract of neonates [14]. The maternal microbiome is believed to influence early microbial colonization [15] and the vaginal and gastrointestinal tracts are considered likely sources of urinary microbiota due to their anatomical proximity [16,17]. Hormonal changes during puberty may further shape the microbiota, though data on how the urobiome develops during childhood is still scarce [13,18].

As in other human body sites, bacteria benefit from the host environment, which provides nutrients, a stable temperature, and limited fluctuations in pH, which is typically mildly acidic (6.0). Yet, the full scope of the advantages that the urobiota offers to the bladder compared to that of the microbiota in other mucosal sites is still much behind and in recent years, the investigations on this topic have increased [19]. As it is well known that microbes play a key role in immune system development post-birth, the urobiota may similarly help prime the immune system and specialized immune cells in the urinary tract [9,20].

Emerging research connects microbial imbalances (dysbiosis) to a range of health issues. Spinal cord injury was associated with microbiota changes in the urinary tract of patients with/without decubitus ulcers [21], while changes in urobiota were reported in patients with lower urinary tract symptoms [22], recurrent urinary tract infections (rUTIs) [23], painful bladder syndromes, and even bladder cancer [24,25]. The impact of gender and age on the urobiome (i.e., the genetic make-up of the urobiota) has been recently reviewed by others [4,19,20]. In general, in the bladder of healthy women, the species belonging to the Lactobacillaceae family (i.e., *Lactobacillus*, *Staphylococcus* and *Gardnerella*) are by far the most abundant and are likely responsible for inhibiting uropathogens growth in the urinary tract by different mechanisms. These include the production of lactic acid which also provides a low-pH environment, the impediment of the attachment of the uropathogens to the mucosal surface, the inhibition of biofilm formation, including through anti-biofilm activity, and the production of hydrogen peroxide. The male microbiota includes *Corynebacterium*, *Streptococcus*, and *Lactobacillus*, though the latter genus is less abundant than in women [26].

The alteration in the microbiome can be regarded as a secondary effect linked to the aging process. As the body undergoes various changes with age, these alterations can influence the environmental conditions of the microbiota, consequently affecting the composition and diversity of commensal microorganisms. In women, in particular, aging is accompanied by hormonal changes, making menopause a significant factor contributing to microbiome changes [27]. As a consequence, the urine of premenopausal women contains an abundant number of *Lactobacillus*, but its prevalence decreases in postmenopausal women. Conversely, abundant *Mobiluncus* species can be seen in postmenopausal women’s urine and this shift is linked with an increased chance of UTIs and the development of rUTIs [28]. The decreased abundance of *Lactobacillus* can be regarded as the primary cause of the above infections because of the lack of their protective effects listed above.

## 2. Bladder Cancer and Influence of Microbial Factors

In the report of the International Agency for Research on Cancer by the WHO, amongst the most frequently diagnosed cancers globally, bladder cancer (BC) ranks ninth for incidence rate and thirteenth for mortality rate. As of 2022, an estimated 614,298 new cases were reported around the world, showing a 7.1% rise compared to 2020. Moreover, recent data show that approximately 1,950,315 people of all genders are living with a BC diagnosis within the last five years, which places BC in the seventh position for prevalence. BC incidence rates are highest in Southern Europe, with countries like Greece having the highest incidence rate among men worldwide, along with Spain, Italy, Belgium, and the Netherlands in Western Europe (https://gco.iarc.who.int/today/en/dataviz/pie?mode=cancer&types=0&sexes=1&populations=900 (accessed on 7 August 2025)). Indeed, when considering gender-specific data, 471,293 new BC cases in men are reported, which accounts for an age-standardized incidence rate of 9.3% of all new cancer recorded among males around the world. This puts BC on sixth place of most diagnosed cancer in men. BC risk increases with the increase in age, and its incidence in men is almost four times higher than in women [29]. It is important to note that, approximately 75% of newly diagnosed BC patients are aged 65+ and approx. 45% are aged 75+, which includes BC as a type of cancer primarily affecting the elderly [30].

As per the guidelines of the U.S. National Comprehensive Cancer Network, BC is categorized into non-muscle-invasive bladder cancer (NMIBC) and muscle-invasive bladder cancer (MIBC) [31]. NMIBC is confined to the urothelium or lamina propria, corresponding to stages 0 and 1, while MIBC involves invasion into the muscle or deeper layers, encompassing stages 2 to 4 [32]. NMIBC shows a good prognosis, although its recurrence rate is high. In contrast to NMIBC, MIBC has a higher risk of metastasis, and its progression is linked to poor prognosis [33]. Epidemiological studies show that although men generally have a higher incidence of BC, women often present with more aggressive and advanced forms of the disease and face worse outcomes [34,35,36].

The leading risk factor for BC is smoking, mainly due to the accumulation of carcinogenic substances like aromatic amines and polycyclic aromatic hydrocarbons in the urine [37]. Such substances are present in tobacco smoke and may cause DNA damage to urothelial epithelial cells and ultimately lead to cancer development [38,39]. Other important risk factors for BC include heavy alcohol consumption and exposure to occupational carcinogens, such as benzidine, 4-chloro-o-toluidine, 4-aminobiphenyl, and 2-naphthylamine, particularly in workers from the rubber and dye industries [40]. Schistosomiasis is also a notable risk factor for BC [41].

It is estimated that globally around 20% of cancers are influenced by microbial factors and that they have a close link with the immune system and a role in tumor cell proliferation as well as in cancer metabolism [42,43,44]. Dysbiosis may decrease the presence of beneficial species involved in proper immune system functioning and maintaining of epithelial cell balance, while increasing harmful species. This imbalance can cause carcinogenesis by affecting mucosal barrier functions and even bacterial translocation to tumor sites. Although the role of the microbiome is an emerging field in BC, experimental evidence is increasing that the microbiome could be a crucial factor in susceptibility to BC [45,46]. However, analyzing these findings is challenging due to the numerous factors that can directly cause change in the composition of the urinary microbiota [47]. Some inconsistent findings have been reported, related to the urobiota in BC patients. Some research show an increased number in bacterial diversity and a differential presence of certain bacterial genera in BC patients compared to non-cancer controls [25]. Chipollini et al. [48] observed a reduction in microbial community in a BC patient’s urine and found specific taxa that were more abundant in BC patients compared to those in general clinic populations such as *Faecalibacterium* and *Bacteroides*. On the other hand, Bi et al. [49] found greater alpha diversity of the urinary microbiota in BC patients and observed more abundance of *Actinomyces europaeus* in these individuals. Furthermore, Bučević Popović et al. [50] did not find major differences in urobiota diversity between 12 cancer patients and 11 healthy controls but did identify a difference in the abundance of specific operational taxonomic units (OTUs) between the two groups. Within BC tissues, an elevated level of phylum Proteobacteria has been seen after multiple studies. Therefore, elevated levels of Proteobacteria could serve as potential markers for dysbiosis in BC diagnosis [51]. On the other hand, genera such as *Ruminococcus*, which are decreased in BC patients, are known for their anti-inflammatory properties and their role in maintaining mucosal homeostasis [52]. A decrease in these health-promoting bacteria may create a more favorable environment for harmful bacteria, triggering inflammation and oxidative stress. The interaction between the tumor and the urobiome could involve the breakdown of normal urothelium, ultimately facilitating the attachment and proliferation of specific microbial taxa [52]. Table 1 provides a summary of studies analyzing the urobiota in BC patients vs. controls (when included). For the sake of consistency, only studies where urine samples were collected and studied are reported. Notably, in three out of sixteen studies, the gender was not reported. Moreover, in studies where female subjects were included, the collection method sometimes involved (i.e., four out of seven studies) the midstream (clean-catch) urine which may add a confounding effect from the possible presence of the vaginal microbiota.

In analogy to other type of cancers, the human urobiota may influence tumorigenesis or facilitate the development of BC through different mechanisms, such as (i) direct DNA damage caused by bacterial toxins (named “genotoxins”), (ii) metabolites from microbiota that may function as potential carcinogens, (iii) bacterial-induced inflammation or biofilm formation that cause inflammation and leads to cancer promotion, and (iv) cellular microenvironment modulation by the microbiome [63,64,65]. For example, colibactin, produced by B2 *Escherichia coli* strains, is a toxin that causes breakage of double-stranded DNA [66]. A recent study shows that *E. coli* lineages producing colibactin increase the prevalence of BC, as well as of colorectal and prostate cancers in some human populations [67]. This study provides the first evidence that the geographical variation in this type of cancer is indeed linked to the increased degree to colibactin exposure. According to Bersanelli et al. [64], the resident urinary microbiota possibly plays a concealed role in BC development. Notably, certain bacterial genera such as *Acinetobacter*, *Stenotrophomonas*, *Staphylococcus*, and *Propionibacterium* are more prevalent in BC patients as compared to healthy controls. In addition, an increased abundance of *Sphingomonas* spp., *Acinetobacter* spp., and *Staphylococcus* spp. can be seen in BC patients [68]. Interestingly a recent study shows that the metabolite indole-3-acetic acid, produced by the beneficial gut bacterium *Parabacteroides distasonis*, significantly decreased in BC patients, exerting tumor-suppressive effects by a receptor mechanism that eventually inhibits BC progression and metastasis [69].

As for inflammation, a study found increased urinary microbiota diversity and inflammatory cytokines in BC patients as well as elevated fatty acids and acylcarnitines, with the latter decreasing after tumor removal [70]. Combined biomarkers from microbiome (Actinomycetaceae), metabolome (arachidonic acid), and cytokines (IL-6) showed high diagnostic accuracy and promise as a noninvasive diagnostic tool.

**Figure 1:**
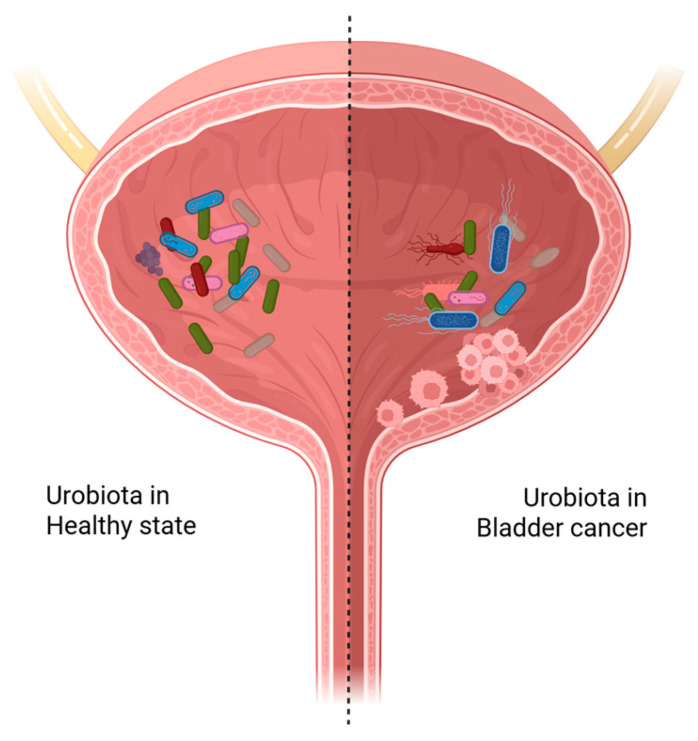
Bladder microbiota in health and cancer. Schematic illustration of the bladder environment in healthy state (left side, lighter) with a diverse and balanced urobiota and cancer state (right side, darker) with an altered (dysbiotic) urobiota containing pathogens that can induce inflammation, damage host DNA, induce biofilm formation, and disrupt the ECM. Created in BioRender. De Biase, D. (2025) https://BioRender.com/w4yvmvp (licensed on 15 October 2025).

As schematically depicted in Figure 1, pathogenic urobiota can attach to the mucosal surfaces of the bladder, leading to either continuous translocation or transient invasions of the bladder tissue [71]. Many bacteria produce proteases that may act both extracellularly and intracellularly and disrupt the normal process of renewal of extracellular matrix (ECM) in the bladder. This disruption can create a modified ECM environment that can promote cancer development [71,72]. For example, the exoenzyme alkaline protease from *Pseudomonas aeruginosa* can degrade ECM components and impair lymphocyte proliferation by inactivating interferon gamma (IFN-ɣ) [73].

The urobiome may not only play a role in the incidence, progression, and recurrence of BC but also in treatment responses. Recent research has highlighted that the role of lactic acid-producing bacteria in BC involves regulating T and NK cell activities, indicating their potential relevance to immune responses [74]. For the cure of high-risk NMIBC, Bacillus Calmette-Guérin (BCG) intravesical immunotherapy is a standard treatment for this purpose [75]. However, there is an incomplete understanding on what the actual mechanism is by which microbiome of urinary tract can influence the response to BCG therapy, but it is said that it is due to the involvement of urobiome’s influence on the mucosal defensin levels. Three types of defensins, which are antimicrobial peptides, are present: human beta defensin 1 (HBD1), which is constitutively produced, and human beta defensins 2 (HBD2) and 3 (HBD3), which are induced defensin. HBD1 is known to protect against BC development, while the urobiome may influence the response to BCG therapy by affecting HBD2 and HBD3 levels [62].

## 3. Culturing and Molecular Techniques to Study the Urobiota

To examine the connections between bacteria in the bladder and host health, it is important to precisely identify bacteria rapidly and at a large scale [76]. Currently, no “gold standard” has been established for the collection, preservation, and storage of urine samples for microbiome research. [77,78]. Various urine collection methods have been used in urobiome research [20], which include first-catch (clean-catch) midstream, transurethral catheterization, and suprapubic aspiration. The latter would be more accurate to assess the microbiome composition of urine, but is invasive and not ethical in control groups. Studies suggest that there are non-substantial differences in microbiota composition between catheter-based urine sample collection and suprapubic aspiration, which makes transurethral catheter-based collection more widely employed in urinary microbiome research [79,80]. Preservation and storage are also of key importance. Typically, refrigeration (+4 °C) is fine in the first hours after collection, but for storage longer than 24 h freezing at −80 °C is of key importance for ensuring the integrity of the microbiome [77,81].

### 3.1. Culture-Dependent: Standard Urine Culture and Expanded Quantitative Urine Culture

Typically, voided urine is used in standard urine culture (SUC) technique as a diagnostic method to confirm rUTIs [82]. This aerobic protocol was mainly designed for culturing *Escherichia coli* to analyze the risk of pyelonephritis during pregnancy [83] and is particularly effective in culturing Enterobacteriaceae at >10^5^ CFU/mL. As described elsewhere [20,84], SUC is performed by inoculating 1 μL of urine onto MacConkey agar plates, then the plates are placed for 24 h at 35 °C under aerobic conditions. However, a likely reason why bacteria were not detected in SUC may be attributed either to the low-biomass of the urobiota, i.e., below the culture threshold of 10^3^ CFU/mL, or to the need of bacteria for different culturing conditions, such as incubation in an increased CO_2_ environment, anaerobic conditions, or extended incubation time [2]. The traditional urine culture procedures were therefore modified by (1) increasing the urine volume for plating, (2) extending the incubation time, and (3) varying the incubation atmospheric conditions. This method is called Expanded Quantitative Urine Culture (EQUC) and it is developed to detect the uropathogens that exist in low abundance, and which were unculturable previously [2,85]. For urine culture in anaerobic condition, 0.1 mL of urine can be used for inoculation onto CDC anaerobe 5% of sheep blood agar (ABAP) plates, then placed under anaerobic conditions for 48 h at 35 °C [2,3,86]. Urine can also be inoculated into pre-reduced broth or Brucella blood agar plates with 5% sheep blood and vitamin K1/hemin supplementation. The plates can then be incubated in an anaerobic chamber with 85% N, 10% CO_2_, and 5% H_2_ at 37 °C [87].

When bacterial counts are below 10 CFU/mL, 1.0 mL of urine can be placed in thioglycolate medium tubes and then incubated aerobically at 35 °C for 5 days [3]. If bacterial growth becomes visible in the thioglycolate medium, the contents can be mixed, and a few drops can be plated on sheep blood agar plate (BAP) and CDC Anaerobe 5% of sheep blood agars for isolation. These plates can be incubated at 35 °C for 48 h under both aerobic and anaerobic conditions.

### 3.2. Culturomics

Culturomics is a culturing approach that employs various culture conditions, matrix-assisted laser desorption/ionization time-of-flight mass spectrometry (MALDI-TOF MS), and 16S rRNA sequencing to identify bacterial species. This high-throughput culture technique initially aimed to create multiple culture environments to support the growth of fastidious bacteria, especially from the human gut. This goal was achieved by enhancing culture media with additives like blood and rumen fluid in blood culture bottles, which facilitated the growth of minority bacterial populations [88]. More recently, culturomics has integrated high-throughput sequencing and culture-dependent methods incorporating microfluidics to isolate and identify new bacterial species. This advancement led to the discovery of a new genus within the Ruminococcaceae family [89]. However, culturomics has notable limitations. It requires significant labor and cannot process as many samples as metagenomics.

Culturomics has been recently applied to the study of the urobiota [90]. The extended culturomics protocol included inoculation of 0.1 mL of urine onto a large plate surface of supplemented BAP and chromogenic agar plates, which is then incubated under aerobic and microaerophilic conditions at 37 °C for 48 h. BAPs can also be incubated under anaerobic conditions at 37 °C for 48 h. Morphologically distinct and representative colonies, after replating, can be identified by using MALDI-TOF MS. Once (and if) the genera are identified by MALDI-TOF, they can be stored as a stock by scraping each colony from the surface of the plate and suspending into 1 mL of liquid media, with the specific media chosen based on the genus identified by MALDI-TOF. After the incubation, 1 mL of liquid culture and 1 mL 50% (*v*/*v*) glycerol are mixed to make the glycerol stocks, which can then be frozen at −80 °C for later use [91]. Despite advancements, culturomics cannot identify ‘not yet culturable’ microorganisms and does not directly yield information on gene expression or bacterial function. Functional insights require genome sequencing of newly isolated species to assess their genetic potential [92].

### 3.3. Culture-Independent: Amplicon Sequencing and Metagenomics

Urine culture remains a routine method for detecting pathogenic microbes; however, it has limitations due to low sensitivity, making it challenging to identify all microbial species through conventional techniques [93]. This drawback is particularly notable for certain microorganisms, especially anaerobes, which may require specific nutrients or supplements and significant time to grow and thus cannot always be cultured effectively, as mentioned in Section 3.2. The advancements in Next-Generation Sequencing (NGS) have revolutionized molecular diagnostics, allowing researchers to quickly access extensive and diverse genetic data such as Amplicon Sequencing and Metagenome Shotgun Sequencing [20], though we should always keep in mind that sequencing does not distinguish live from dead bacteria [79].

Amplicon sequencing, a PCR-based technique, focuses on marker genes, such as the 16S rRNA subunit, with nine hypervariable regions (V1–V9). These regions facilitate the measurement of evolutionary distances among bacterial species and provide conserved inter-regional sequences essential for primer design. This technology not only identifies bacterial species but also assesses their diversity and quantifies interrelationships within a single sample. This intra-sample diversity is referred to as alpha diversity, in contrast to beta diversity, which represents differences across multiple samples [94]. Although urinary microbiome research has advanced, 16S rRNA amplicon sequencing often limits resolution to the family or genus level, making species-level identification more challenging, especially in complex polymicrobial samples, and it cannot assess functional genes within the microbial community [95]. Furthermore, viruses, bacteriophages, and fungi cannot be identified.

To analyze all the genetic material inside a (urine) sample, metagenomics, a culture-independent, high-throughput sequencing technique is used. Metagenomics enables the characterization of complete genomes within the genetic pool, achieving higher taxonomic and functional resolution. Unlike other approaches that rely on specific genetic markers, metagenomics offers an unbiased, in-depth examination of the microbiome [9]. Metagenomics can be helpful in community profiling and can improve understanding of antibiotic resistance or virulence genes associated with uropathogens [96,97,98] and provides detailed community composition analysis. Many novel uropathogens have been identified by metagenomics such as *Ureaplasma*, *Alloscardovia*, and *Actinotignum*, which are typically present in low abundance within the urinary tract [99]. Although sequencing the entire DNA content has clear advantages, it generally requires high sequencing depth for effective de novo assembly, especially in complex microbial communities. NGS offers significant benefits, providing a broad overview of the urinary microbiome and eliminating the need for time- and labor-intensive culturing and species isolation steps. This capability has facilitated dynamic, large scale, and comprehensive analyses, allowing for the detection of microbes that are challenging to culture [2].

**Figure 2:**
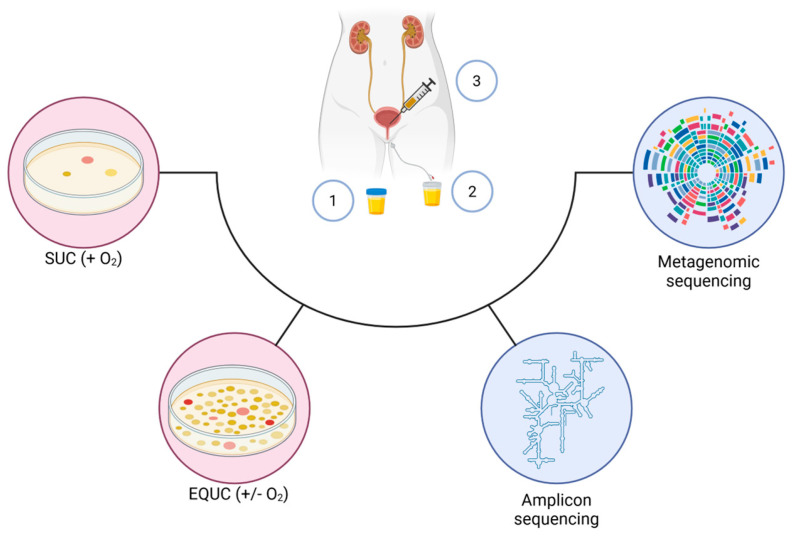
Culturing and molecular techniques to study the urobiota. The urine collection methods include clean-catch midstream (1), transurethral catheterization (2), and suprapubic aspiration (3). Culturing methods include SUC (standard urine culture using 1 µL of urine, carried out in aerobic conditions) and EQUC (expanded quantitative urine culture using 100 µL of urine, carried out in both aerobis and anaerobic conditions). Created in BioRender. De Biase, D. (2025) https://BioRender.com/buwvgw3 (licensed on 29 October 2025).

Figure 2 summarizes most of the key techniques described above, from sample collection methods to culturing methods and sequencing.

### 3.4. Animal Models and 3D Organotypic In Vitro Model

For investigating interactions between hosts and microbiota in vivo, animal models have been used as an experimental tool. However, some challenges have restricted the use of animal models such as high costs, limited reliability, and ethical concerns. Furthermore, animal models cannot fully replicate the intricate host–microbe interactions as in the human body due to variations in factors such as genetics, anatomy, diet, physiology, and life cycles of model animals and humans [100]. Despite these limitations, in the case of BC animal models, N-butyl-N-(4-hydroxy butyl) nitrosamine (BBN)-induced has been established [101]. More recently, a novel mouse model of upper urinary tract urothelial carcinoma (UTUC) was obtained by treating multiple mice strains, of different sexes, with BBN: the non-engineered UTUC mouse model (only female BALB/c mice) was demonstrated to reflect human UTUC in many molecular aspects [102]. Notably, the low relative abundance of *P. distasonis* in the gut was reverted with a dietary intervention, which consisted mainly of removing alanine: the suppression of the TNF-related inflammatory gene expression in the upper urinary tract was observed even in the presence of BBN. The reduced relative abundance of *P. distasonis* and its link to urothelial carcinoma is likely associated to the decreased levels of its metabolite indole-3-acetic acid [69], as described in Section 3.

As for in vitro studies, these often depend on primary cells (two-dimensional or 2D cell culture) and monolayer cultures of cell lines, which investigate tissue function and disease pathogenesis, including infections, cancer, and metastasis. However, these 2D and monolayer cultures have limitations in capturing the intricate influence of the stromal environment, which is crucial for the development of diseases. Three-dimensional (3D) organotypic models offer a promising approach to gain a deeper understanding of molecular disease development, moving closer to in vivo conditions [103,104]. An array of 3D organotypic models has been established, in order to study also the interaction between bacteria and microbiome [105,106]. Also in the urological field of cancer, organoid technology is witnessing significant advancements [107] and 3D bio-printed in vitro models of the bladder and urethra have been developed [108,109]. However, the literature is still scarce in this area of research.

## 4. Gut–Bladder Axis in Bladder Cancer

The interplay between the gut and the bladder microbiota (the so-called “gut–bladder axis”) is still an emerging field of research, including the study of the implication of gut microbiota (GM) and dysbiosis as an etiological factor in BC. As mentioned in Section 2, BC is a cancer that more commonly affects a population aged 65+, that is more at risk of dysbiosis in the gut and in the bladder because of aging.

A two-sample Mendelian randomization (MR) analysis predicted that the genus *Bifidobacterium*, the phylum, and class Actinobacteria and the *Ruminococcus torques* group in GM were associated with an increased risk of BC, while *Allisonella* was associated with a decreased risk [110]. Another two-sample Mendelian randomization study showed a significant a causal relationship between *Bilophila* and BC, while *Oscillibacter*, Ruminococcaceae NK4A214 group, and Enterobacteriales were protective against BC [111]. Whether the observed associations can be assigned to H_2_S production by *Bilophila* (a known genotoxic compound) and SCFA production by the protective species still remain to be demonstrated. A Mendelian randomization study of GM showed that the family Pasteurellaceae, *Eubacterium coprostanoligenes* group, and the order Pasteurellales significantly increase the risk of BC. Conversely, the genus *Escherichia Shigella* was associated with a decreased risk [112].

In one of the first studies where the urinary microbiota and GM of patients with BC were compared with a healthy control group, class Alphaproteobacteria, order Rhodospirillales, order Flavobacteriales, and family Flavobacteriaceae were specific only in females with BC [62]. A difference in the abundance of phylum Desulfobacterota across tumor grade was also observed, i.e., most abundant in Grade G1 and least in G2. However the study had limitations, i.e., small groups with diverse history (sociodemographic, BCG therapy, smoking), single-centered study, and collection of urine by midstream [62].

A study involving n. 142 NIMBC patients and n. 48 controls demonstrated that the GM in BC patients had a higher prevalence of *Prevotella* and *Porphyromonas* but a reduced abundance of *Faecalibacterium* compared with controls [113]. The alpha diversity indices in the BC group increased, while no statistically significant differences in abundances of the above bacteria were observed between genders. In the same study, the differences in the composition of the GM between patients who did not respond to the neoadjuvant chemotherapy (n. 57) vs. controls showed higher levels of *Bacteroides* and *Pseudomonas* compared to the controls, while Lachnospirenaceae (producers of SCFAs) were more abundant in responders [113]. A more direct association between a specific bacterium *P. distasonis* and BC was provided in a study where the GM of n. 50 BC patients were compared to that of n. 22 matched controls: the genus *Parabacteroides* was more abundant in the controls [114]. Notably, in the same study, it was shown that the combination of *P. distasonis* and anti-PD-1 monoclonal antibody significantly inhibited tumor growth and reduced its weight in bladder tumor-bearing mice, compared to treatment with anti-PD-1 antibody alone. Immunohistochemistry and RNA-sequencing showed that *P. distasonis* promoted anti-tumor immune responses by potentiating the efficacy of anti-PD-1 therapy through immune activation and modulation of tumor-related signaling pathways [114].

While the objective of this section was mostly on the potential of microbial players of the GM on the development of BC, the role of inflammation should also be taken into account. This topic is investigated [70] and has been recently reviewed [9,115].

**Figure 3:**
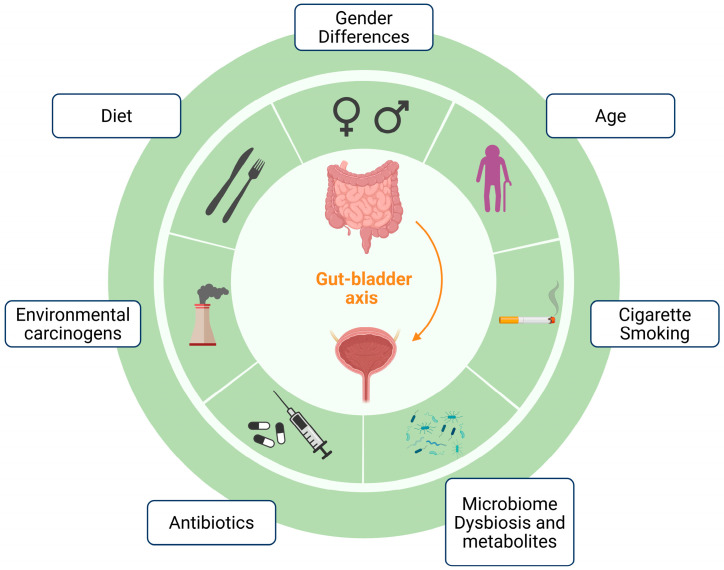
Contributing factors to bladder cancer. Created in BioRender. De Biase, D. (2025) https://BioRender.com/17jtfaq (licensed on 31 August 2025).

Figure 3 provides a graphical overview of the factors contributing to BC, dealt with in this and previous sections, and in the next upcoming section.

## 5. Gut-Derived Microbial Metabolites and Bladder Health

Our understanding of the gut–bladder axis with respect to the effect of microbial metabolites is still limited, i.e., the connection between GM, gut and/or bladder metabolites of microbial origin, and BC is still embryonic. This is confirmed by recent reviews dealing with the impact of dietary metabolites, such as SCFAs (important in regulating the function of the epithelial as well as the mucosal barrier and systemic immunity), hardly mentioning the bladder in their analysis [116,117,118]. Given that a good level of understanding is available on SCFAs and that this topic has been covered by the above studies, herein we focus on other metabolites that may potentially affect bladder health especially in BC. However, it is important here to mention that low levels of butyrate have been detected in the BC patients [119]. Butyrate helps in the proliferation of intestinal epithelial cells and helps in maintaining the mucosal integrity: its reduced levels compromise the epithelial homeostasis, ultimately diminishing its inhibitory effects on BC progression. The inhibitory effect is mediated by the host G-protein-coupled receptors. Furthermore, butyrate can enter cells via host plasma membrane transporters and inhibits histone deacetylases inside the host cells, which leads to histone hyperacetylation and changes in gene expression, eventually leading to lowering of tumor cell proliferation and increased apoptosis [118]. It is well known that SCFAs are by-products of fermentation, mostly produced in the colon, by GM members that degrade and retrieve energy from undigested dietary fibers [116]. Diet can therefore provide a strategic approach to prevent or treat BC patients. This is the hypothesis that guided the work by Then et al. [120] who investigated the ability of high-fiber diets in sensitizing BC to irradiation. They used different dietary regimens at the same time in which mouse bladder tumor cells were inoculated subcutaneously in the allograft immunoproficient mouse model; these dietary regimens included either normal chow or 0.2% cellulose, or psyllium, or psyllium + resistant starch (RS, known to contain fibers that produce butyrate), or psyllium + inulin (a fiber readily fermentable and known as radiosensitiser). Psyllum is a dietary fiber used to reduce the side effects of radiotherapy [121]. Psyllium + RS as well as psyllium + inulin significantly delayed tumor growth compared to the control group (i.e., 0.2% cellulose). Furthermore, when bladder tumors were irradiated, psyllium + RS significantly radiosensitized the tumors compared to 0.2% of cellulose and psyllium alone. The study also showed that the GM modified by the dietary fiber regimen might be required to activate immune responses at a systemic level. Overall, the study confirms that the use of dietary fibers by increasing SCFAs affects the outcome of the bladder tumors. This is an effect of the shaping of the GM in favor of an increase in the relative abundance of the Lachnospiraceae family, and of *Bacteroides* genus abundance [120].

Amongst the microbial-derived metabolites, we should distinguish those that originate from the GM and then reach the bladder from those that are produced inside the bladder. Herein, we review some very recent reports on both aspects.

Amongst metabolites of GM origin, the metabolite 3-IAA, mentioned in Section 2 and Section 3.4 above, has been recently analyzed in great detail by Li et al. [69]. In addition to demonstrating that the increased abundance of *P. distasonis* in GM is associated with an improved prognosis in BC patients, the authors provided evidence that specified that the metabolite 3-IAA derived from *P. distasonis* has a tumor-suppressive effect and inhibited BC cells migration in a dose-dependent manner. Given that 3-IAA is a metabolite originating from the amino acid tryptophan, the derivatives of which are known to bind the AhR receptor, which are activated in tumor-associated macrophages, the study demonstrated first of all that 3-IAA binds AhR and, secondly, that 3-IAA exerts its tumor-suppressive effect by activating AhR (as demonstrated by knocking down the AhR gene). The intracellular effect consists of a reduced expression of fatty acid synthase and stearoyl-CoA desaturase. The former enzyme in particular is highly expressed in BC patients, who show a worse overall survival [69]. As a consequence of the decreased expression of fatty acid synthase, the incorporation of polyunsaturated fatty acids in membrane phospholipids increases and this ultimately causes an increase in cellular sensitivity to ferroptosis.

On the other hand, some metabolites originate from the metabolism of molecules that are recognized as environmental and occupational carcinogens and represent major risk factors in BC (see Section 2). Amongst these are N-butyl-N- (4-hydroxybutyl)-nitrosamine (BBN), a nitrosamine compound, as well as other organic contaminants related to it, that have been described to induce BC [122,123]. Using a mouse model, Roje et al. [123] provide evidence that GM may enhance the development of BC in BBN-exposed mice and that a decreased bacterial load has a positive effect, i.e., the reduced metabolism by the GM of BBN into BCPN, which is its oxidation product, leads to decrease in the concentration of BCPN in the urine, from which it is eliminated, thereby decreasing the urothelial exposure to this carcinogen by altering its pharmacokinetics [123]. The identification of specific genera in both mouse and human GM suggests that interpersonal differences could play a role in individual predisposition to tumor development.

Xenobiotics transformation can be carried out not only by the GM, but also by the microbiota in other body sites, including by bladder. This is extremely important when we consider studies that have demonstrated that out of 212 pharmaceuticals, only one third is excreted through the feces, with the majority through urine. In some cases, paracetamol, acetylsalicylic acid, and gabapentin (an antiepileptic drug) excretion is performed almost entirely through the urine [124]. Furthermore, xenobiotics are exposed in the gut to the GM, which is much denser than the urobiota and also the exposure to xenobiotics in the gut is much longer (55 h) than in the bladder (4 h). Also, oxygen levels are different in the two niches. Using a genome mining approach, Marti et al. [125] analyzed the distribution of enzyme classes in the urobiota, in particular from genomes of bacteria isolated using EQUC from women samples [84]. The analysis indicated that the distribution of enzyme classes is discontinuous even within species in the same genus and that urinary *Gordonia* and *Bacilli* have a broad biotransformation potential. Amongst the EC classes, the oxidoreductases (in particular, NADP-dependent oxidoreductases/dihydropyrimidine dehydrogenases EC 1.3), transferases (EC 2), and amidases, amongst the hydrolases (EC 3), were the most represented in the urinary bacterial genomes.

## 6. Conclusions and Future Directions

The study of the urobiota and the urobiome in the urine of BC patients is a field of merging interest, especially in the last 15 years since the long-lasting belief about urine sterility above the urethra has been demonstrated to be untrue. We focused on studies, summarized in Table 1, that analyzed the urobiota using urine specimens from the BC group vs. control (i.e., healthy subjects) in the last 10 years. While Actinobacteria Bacteroidetes, Firmicutes, and Proteobacteria were found as the most abundant phyla in both groups, and in most of the studies, no clear signature of BC or healthy state has been demonstrated to date. This is because the studies were limited in size of recruited patients/controls, urine collection mode, gender, stage of BC, and lifestyle. A good knowledge of the microbial metabolism and enzymatic activities that take place in the bladder is extremely relevant, also in terms of protection from cancer.

Notably, two recent studies [69,114] demonstrate the therapeutic potential of *P. distasonis*, which is a producer in the gut of 3-IAA that is a metabolite of the amino acid tryptophan, that has a tumor-suppressive effect, inhibiting BC cell migration in a dose-dependent manner. Furthermore, when *P. distasonis* is delivered in combination with alpha-PD-1 mAb, it is effective in improving the effect of anti-PD-1 immunotherapy, likely by activating immune- and anti-tumor-related pathways. The two findings might be linked.

The observed decrease in beneficial microbial metabolites, particularly butyrate, in BC patients underlines a potentially important role of SCFAs in bladder mucosal immunity, epithelial integrity, and tumor suppression. Exploring microbiota-based SCFA restoration therapies may open unique pathways for BC prevention as well as for noninvasive therapy. The study by Then et al. [120] supports the approach of high-fiber diets in radiotherapy. In general, diet-based interventions and probiotic supplementation is a field of great promise.

The study of the relationship between the bladder microbiome and the gut microbiome is still preliminary and requires comprehensive effort to uncover the full impact of these hidden and overlooked microbial communities. As for the case of *P. distasonis*, as research in this field continues to evolve, it will open doors to potential therapeutic interventions, diagnostic biomarkers, and a deeper understanding of BC treatment and recurrence.

## Figures and Tables

**Table 1:** Summary of urobiota studies using urine specimens in BC patients ^(a)^.

Study	Sample Size (Cancer/Healthy)	Gender (Male/Female)	Cohorts and Diversity	Abundance and Key Findings	Conflicting Findings
Xu et al. [53]	8/6	not specified	- Cancer vs. healthy. - Higher average of number of genera in cancer patients.	- *Acinetobacter* was the most abundant in both groups. - *Streptococcus* was dominant in five of the eight cancer samples. - *Pseudomonas* or *Anaerococcus* were dominant in two cancer samples with low *Streptococcus*.	- A small and early study. Findings may reflect small sample size or lack of control for gender.
Bucevic Popovic et al. [50]	12/11	only male	- Cancer vs. healthy. - No significant difference between the groups.	- Firmicutes, Actinobacteria, Bacteroidetes, and Proteobacteria were the most abundant in both groups. - *Fusobacterium* was higher in the cancer group. - *Corynebacterium*, *Streptococcus*, and *Veillonella* were more abundant in the healthy group.	- Though *Fusobacterium* was higher in cancer group, no major diversity differences were found. - No later studies aligned completely with these findings, suggesting these results may depend on population differences.
Wu et al. [25]	31/18	only male	- Cancer vs. healthy. - A significant difference between the groups.	- Proteobacteria, Firmicutes, Actinobacteria, and Bacteroidetes were the most abundant in both groups. - *Acinetobacter*, *Anaerococcus*, and *Rubrobacter* were more abundant in the cancer group. - *Serratia*, *Proteus*, and *Roseomonas* were more abundant in the healthy group.	- Reported higher number of *Acinetobacter* in cancer group, which conflicts with the studies, showing unchanged diversity. - All the controls were male and with various non-neoplastic conditions, such as renal cyst.
Bi et al. [49]	29/26	not specified	- Cancer vs. healthy. - A significant difference between the groups.	- Firmicutes, Actinobacteria, Proteobacteria, and Bacteroidetes were the most abundant in both groups. - *Actinomyces europaeus* was higher in the cancer group. - *Streptococcus*, *Bifidobacterium*, *Lactobacillus*, and *Veillonella* were more abundant in the healthy group.	- This study identified *Actinomyces europaeus* as a potential biomarker for BC, however larger studies are required to confirm this finding.
Mai et al. [54]	24 */0	18 male 6 female	- Cancer patients only.	- *Lactobacillus*, *Streptococcus*, Enterobacteriaceae g., *Ureaplasma*, *Stenotrophomonas*, *Staphylococcus*, *Enterococcus*, and *Corynebacterium* were the most abundant genera in the cancer group.	- Analyzed just cancer patients only. Absence of a control group limits comparison with other studies, and it becomes difficult to determine whether these bacteria are cancer-specific.
Moynihan et al. [55]	33/8	only male	- Cancer vs. healthy. - Smokers vs. non-smokers. - Alpha diversity: No significant differences (smoking and cancer vs. no cancer). - Beta diversity: No significant differences (smoking and cancer vs. no cancer).	- *Lactobacillus*, *Turicibacter*, and *Bacteroides* were the most abundant genera overall. - No taxa were significantly enriched or depleted by smoking status or by cancer status.	- Did not find any major microbiota differences between the two groups. The lack of differences may be due to small sample size of controls and specific patient population.
Mansour et al. [56]	10 **/0	5 male 5 female	- Cancer patients only. - No significant differences in alpha diversity.	- Firmicutes, Proteobacteria, Actinobacteria, Cyanobacteria, and Bacteroidetes were the most abundant phyla. - *Lactobacillus*, *Corynebacterium*, *Streptococcus*, and *Staphylococcus* were the most abundant genera (with age/gender differences across samples).	- Small sample size, as patients whose tissue samples were analyzed were excluded in this table. Analyzing only one group makes it difficult to compare and interpret the results.
Pederzoli et al. [57]	49 */59 *	Patients: 36 male 13 female Controls: 34 male 25 female	- Cancer vs. healthy. - No significant differences in alpha and beta diversity (BC and controls), except beta diversity between female cohorts. - Higher bacterial load in cancer samples (both genders).	- Proteobacteria, Firmicutes, and Bacteroidetes were the most abundant phyla overall. - Females with BC: Enriched with *Klebsiella*. - Males with BC: Enrichment of order Opitutales, family Opitutaceae, and class Acidobacteria-6.	- This study and that of Mansour et al. [56] both confirmed that the microbiota of urine differs between cancer and control groups, but the type of difference (diversity vs. composition) varies between the studies. - These changes may be due to gender and geographical differences.
Zeng et al. [46]	62/19	only male	- Cancer vs. healthy. - Bacterial richness (Observed Species index, Chao1 index, Ace index) was significantly increased in the cancer group. - No significant differences in the Shannon and Simpson indexes between the two groups.	- *Anoxybacillus*, *Geomicrobium*, *Massilia*, *Micrococcus*, *Thermomonas*, *Nocardioides*, *Larkinella*, *Jeotgalibacillus*, and *Brachybacterium* were increased in the recurrence group. - *Lactobacillus* was higher in the non-recurrence group.	- Found higher bacterial diversity in cancer groups, like Wu et al. [25] but conflicts with studies that showed reduced or unchanged diversity such as Chipollini et al. [48] and Moynihan et al. [55]. - These changes may be due to differences in patient gender and disease stage.
Chipollini et al. [48]	25/10	not specified	- Cancer vs. healthy. - No significant difference in alpha diversity between the groups. - A significant difference in beta diversity between the two groups.	- *Bacteroides* and *Faecalbacterium* were higher in cancer group. - *Bacteroides*, Burkholderiaceae, and *Lachnoclostridium* were higher in healthy group. - Significantly lower microbial diversity in the cancer group compared to the healthy group.	- No specific genus consistently associated with tumor grade or stage. - Suggested that loss of bacterial diversity may be linked to carcinogenesis or inflammation, which contrasts with studies reporting higher or unchanged diversity. - These changes may be due to variations in population and gender composition.
Hourigan et al. [58]	22 */**/0	14 male 8 female	- Midstream urine vs. cystoscopy. - No differences in alpha and beta diversity by collection method. - In males, voided vs. cystoscopy samples from the same individual showed differences.	- *Stenotrophomonas* was more abundant in cystoscopy samples. - *Tepidomonas* was more abundant in males. - *Prevotella* and *Veillonella* were more abundant in females.	- This tells why studies with different sampling methods show conflicting results.
Hussein et al. [59]	43 **/10 *	Patients: 36 male 7 female Controls: 5 male 5 female	- Cancer vs. healthy. - No significant difference in alpha diversity between the groups. - A significant difference in beta diversity between the two groups.	- *Brevibacterium*, *Achromobacter*, *Actinomyces*, and *Brucella* were abundant in cancer group.	- Observed lower bacterial diversity in BC urine compared with controls. - The number of control samples is much lower than the number of cancer samples, making a fair comparison difficult.
Ma et al. [60]	15/11	only male	- Cancer vs. healthy. - Smokers vs. non-smokers (within cancer and controls). - Cancer vs. healthy: No significant difference in alpha diversity between the groups. - A significant difference in beta diversity between the two groups. - Smokers vs. non-smokers: Significantly higher alpha diversity in smoking BC compared to non-smoking BC. - A significant difference in beta diversity between these two groups.	- *Stenotrophomonas*, Enterococcaceae, *Enterococcus*, *Myroides*, and *Parvimonas* were abundant in the cancer group. - Family_XI, Clostridiaceae_1, *Sphingomonas*, Deltaproteobacteria, and Gemmatimonadetes were higher in the healthy group.	- Found that smoking affected urinary microbiomes and may contribute to cancer risk or progression. - Further studies with smoking status as a variable are required to have a better insight into how smoking influences the urobiome.
Oresta et al. [52]	51/10	only male	- Cancer vs. healthy. - Alpha diversity: Evenness was higher in BC vs. controls. - Beta diversity: No significant difference in beta diversity between the two groups; high inter-individual heterogeneity.	- Phyla Firmicutes, Actinobacteria, Bacteroidetes, and Proteobacteria were abundant in both groups. - *Veillonella* and *Corynebacterium* were abundant in the cancer group. - *Ruminococcus* and an unclassified genus of Enterobacteriaceae decreased in the BC group.	- The results contrast with the studies, showing increased or decreased diversity, which may be explained by differences in sampling method and cancer stage. - The number of control samples is much lower than the number of cancer samples, making a fair comparison difficult.
Qiu et al. [61]	6 */4 *	Patients: 5 male 1 female Controls: 3 male 1 female	- Urinary tract tumors vs. healthy. - A significant difference between the groups.	- Higher abundance of *Finegoldia* and *Varibaculum* in patient group. - Both *Finegoldia* and *Varibaculum* were positively correlated with urine pH.	- A very small study that lacks sufficient numbers of controls and patients for comparison with other studies.
Chorbinska et al. [62]	18 */7 *	Patients: 14 male 4 female Controls: 5 male 2 female	- No significant difference in alpha and beta diversity between the groups.	- *Lactobacillus* was more common in patients with a history of Bacillus Calmette–Guérin (BCG) therapy. - Genus *Howardella* and a strain *Streptococcus anginosus* were more common in female patients.	- The lack of significant differences contrasts with many other studies, possibly due to small sample size.

^(a)^ Only in those studies where female patients/controls were included, the collection methods is provided because this may provide an explanation to some of the differences in the findings: * midstream (clean-catch) urine; ** cystoscope (during transurethral resection of a bladder tumor).

## Data Availability

No new dataset(s) were created as part of this work. All the relevant resources were duly cited.

## Abbreviations

The following abbreviations are used in this manuscript:

2D	Two-dimensional
3D	Three-dimensional
ABAP	Anaerobe 5% sheep blood agar
BAP	(Sheep) blood agar plate
BC	Bladder cancer
BCG	Bacillus Calmette-Guérin
ECM	Extracellular matrix
EQUC	Expanded Quantitative Urine Culture
GM	Gut microbiota
MALDI-TOF MS	Matrix-Assisted Laser Desorption Ionization-Time of Flight Mass
MIBC	Muscle-invasive bladder cancer
NGS	Next-Generation Sequencing
NMIBC	Non-muscle-invasive bladder cancer
rUTIs	Recurrent urinary tract infections
SCFAs	Short-chain fatty acids
SUC	Standard urine culture
UTIs	Urinary tract infections
UTUC	Urinary tract urothelial carcinoma
