# Correction: Butt, U.A.; De Biase, D. The Urinary Microbiota and the Gut–Bladder Axis in Bladder Cancer. *Int. J. Mol. Sci.* 2025, *26*, 10558

**DOI:** 10.3390/ijms27031521

**Published:** 2026-02-04

**Authors:** Usman Akhtar Butt, Daniela De Biase

**Affiliations:** Department of Medico-Surgical Sciences and Biotechnologies, Sapienza University of Rome, Corso della Repubblica 79, 04100 Latina, Italy; u.akhtarbutt@gmail.com

In the original publication [1], there are two references that were retracted, which need to be corrected.

17. Dubourg, G.; Morand, A.; Mekhalif, F.; Godefroy, R.; Corthier, A.; Yacouba, A.; Diakite, A.; Cornu, F.; Cresci, M.; Brahimi, S.; et al. Deciphering the Urinary Microbiota Repertoire by Culturomics Reveals Mostly Anaerobic Bacteria From the Gut. *Front. Microbiol.* **2020**, *11*, 513305. https://doi.org/10.3389/fmicb.2020.513305.
63. Poore, G.D.; Kopylova, E.; Zhu, Q.; Carpenter, C.; Fraraccio, S.; Wandro, S.; Kosciolek, T.; Janssen, S.; Metcalf, J.; Song, S.J.; et al. Microbiome analyses of blood and tissues suggest cancer diagnostic approach. *Nature* **2020**, *579*, 567–574. https://doi.org/10.1038/s41586-020-2095-1.

With this correction, the order of some references and relevant statements has been adjusted accordingly. [Retracted Paper 1]—Deciphering the Urinary Microbiota Repertoire by Culturomics Reveals Mostly Anaerobic Bacteria From the Gut [17] in the accepted article. The scientific content of [17] could not be replaced by any other publication, as far as the authors know. Therefore, the authors decided to remove any mention of the “gastrointestinal tract” (specifically referring to [17]), but included [84] (now [17]), which is equally relevant on the interconnection of bladder–vagina microbiomes.

Original sentence in the article: “…… [15] and the vaginal and gastrointestinal tracts are considered likely sources of urinary microbiota due to their anatomical proximity [16,17]”. Now rephrased as follows: “…… [15]. Furthermore, in females the bladder and vagina harbor microbial communities with several bacterial taxa commonly detected at both sites, likely due to their anatomical proximity [16,17].”

[Retracted Paper 2]—Microbiome analyses of blood and tissues suggest cancer diagnostic approach ([63] in the accepted article).

The text does not need any changes. The authors removed [63] and added [66] (now [65]), which is equally relevant in that sentence.

The authors state that the scientific conclusions are unaffected. This correction was approved by the Academic Editor. The original publication has also been updated.
